# Recent Trends and Advances of Co_3_O_4_ Nanoparticles in Environmental Remediation of Bacteria in Wastewater

**DOI:** 10.3390/nano12071129

**Published:** 2022-03-29

**Authors:** Anuoluwapo Anele, Sherine Obare, Jianjun Wei

**Affiliations:** 1 Department of Nanoscience, Joint School of Nanoscience and Nanoengineering, University of North Carolina at Greensboro, Greensboro, NC 27401, USA; aoanele@uncg.edu; 2 Department of Nanoengineering, Joint School of Nanoscience and Nanoengineering, North Carolina A&T State University, Greensboro, NC 27401, USA

**Keywords:** anti-bacterial agents, bacteria, drug resistance, antibiotics, antibiotic resistance, antibacterial mechanism, cobalt oxide nanoparticles, environment, wastewater, remediation

## Abstract

Antibiotic resistance is a formidable global threat. Wastewater is a contributing factor to the prevalence of antibiotic-resistant bacteria and genes in the environment. There is increased interest evident from research trends in exploring nanoparticles for the remediation of antibiotic-resistant bacteria. Cobalt oxide (Co_3_O_4_) nanoparticles have various technological, biomedical, and environmental applications. Beyond the environmental remediation applications of degradation or adsorption of dyes and organic pollutants, there is emerging research interest in the environmental remediation potential of Co_3_O_4_ nanoparticles and its nanocomposites on antibiotic-resistant and/or pathogenic bacteria. This review focuses on the recent trends and advances in remediation using Co_3_O_4_ nanoparticles and its nanocomposites on antibiotic-resistant or pathogenic bacteria from wastewater. Additionally, challenges and future directions that need to be addressed are discussed.

## 1. Introduction

Cobalt oxide (Co_3_O_4_) is a p-type semi-conducting material and a transition metal oxide [1]. It is antiferromagnetic and possesses a spinel crystal structure [2]. Co_3_O_4_ nanoparticles also have optical bands between 1.48–2.19 eV, within which Co_3_O_4_ can be used as a photocatalyst when excited with visible light [2]. Cobalt occurs in two oxidation states that are readily available, namely Co^2+^, Co^3+^ [3], and Co^4+^ [3,4], making it attractive for several industrial applications [4]. Cobalt oxide is abundant in nature, with Co_3_O_4_ being the most stable form [5]. Other forms of cobalt oxide are cobalt (II) oxide (CoO) and cobalt (III) oxide [6]. In this review, our focus is on cobalt (II, III) oxide (Co_3_O_4_). Co_3_O_4_ nanoparticles have been used for applications such as energy storage, solar cells, capacitors, gas sensors, field emission materials, magneto-resistive devices, field effect transistors, and rechargeable Li-ion batteries [2,7]. They have also been used as photocatalysts for the degradation or adsorption of dyes and organic pollutants, as well as antimicrobial, antioxidant, and anticancer applications [2,7,8,9,10,11,12,13,14,15]. The remediation potential of Co_3_O_4_ nanoparticles is encouraging, since they are efficient in degrading pollutants that they have an affinity for and are also alternatives to the more expensive noble metals [1]. Although not as popular as the noble metals, some metal nanoparticles, and other metal oxide nanoparticles, Co_3_O_4_ nanoparticles are promising for the remediation of antibiotic-resistant and/or pathogenic bacteria in wastewater. Excellent reviews exist that address the biomedical [2,5] and catalytic applications [2] of Co_3_O_4_ nanoparticles, which cover applications of Co_3_O_4_ nanoparticles with respect to dye degradation, treatment of malignant cells, antimicrobial activity, and anti-proliferative activity on cancer cells.

Antibiotic resistance is a concerning global environmental and health threat. The discovery and use of antibiotics quickly led to relief from certain diseases. However, factors such as the misuse of antibiotics for clinical and animal production uses [16,17,18,19], global migration, and selection pressure of microbes [17] contributed to the increased incidence of antibiotic resistance in society. The role of the environment [20] in the incidence of antibiotic resistance has also been recognized. For example, wastewater treatment methods do not eliminate all antibiotic-resistant bacteria or antibiotic-resistant genes in wastewater [21,22]. Thus, there has been an impetus for investigating alternative methods for combating antibiotic-resistant bacteria and particularly genes in wastewater [23,24,25]. Nanoparticles may destroy bacteria membranes, gain access to cellular content, and inflict further damage [26,27]. Metal oxides [27], including Co_3_O_4_ nanoparticles [2,5], have antibacterial or inhibitory effects on bacteria. Increased attention is being given to Co_3_O_4_ nanoparticles in this regard due to their antibacterial effect and availability. However, there is a lack of a comprehensive review on nanoparticles in wastewater bacteria remediation applications, specifically the potential of Co_3_O_4_ nanoparticles for bacteria remediation applications, to the best of our knowledge. Therefore, this review examines the research progress that has been made in recent years with respect to using Co_3_O_4_ nanoparticles for the remediation of antibiotic-resistant and/or pathogenic bacteria in wastewater. In addition, we discuss current challenges of using Co_3_O_4_ nanoparticles for bacteria remediation in wastewater treatment and an outlook of future research directions.

## 2. Synthesis

**Figure 1:**
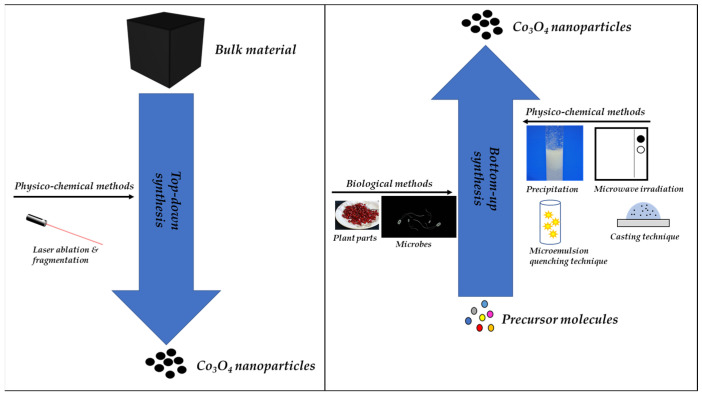
Overview of synthesis methods for cobalt oxide (Co_3_O_4_) nanoparticles. Physicochemical methods such as laser ablation [32,33], laser fragmentation [34], casting technique [35], precipitation [36,37], microemulsion quenching technique [38], and microwave irradiation [36,39] have been used for synthesis. Biological methods based on plants [1,7], biological molecules [40,41], and microbes [3] have also been applied in the synthesis of Co_3_O_4_ nanoparticles.

The synthesis of nanoparticles can be carried out using different methods broadly split between a top-down and a bottom-up approach (Figure 1). Top-down approaches, as the term suggests, are methods of synthesizing nanoparticles using bulk materials as the starting material, which is then broken down into desirable smaller forms [28]. This method is simple and retains the original integrity of the bulk material, although surface structure and crystallographic imperfections are possible [29]. On the other hand, bottom-up approaches “build” the nanoparticles using smaller molecules as the starting material or building blocks [28,29]. The building blocks for the bottom-up synthesis of nanoparticles are atoms, molecules, and other particles that are miniature compared to the previous two [29]. The top-down approach is destructive, whereas the bottom-up approach is characterized by construction [28,30]. Hence, the top-down approach can be described as “synthesis by destruction”, and the bottom-up approach described as “synthesis by construction”. The synthesis of nanoparticles can also be conducted using biological, chemical, or physical approaches. These specific methods fall under either top-down or bottom-up approaches of synthesis. Biological methods and some chemical methods are bottom-up approaches of synthesis, and some chemical methods, particularly physical or mechanical methods, are top-down approaches of synthesis [29,31].

### 2.1. Physicochemical Methods

Several physicochemical methods for synthesizing cobalt oxide nanoparticles have been reported in the literature, with some select examples discussed here (Table 1). The casting technique was used to synthesize cobalt oxide nanoparticles using hydroxyl cellulose and CoCl_2_·6H_2_O as precursors, followed by calcination, to yield nanoparticles that were 15 nm in size [35]. In the casting technique, the precursors in the solution are applied to an appropriate surface, such as glass or stainless steel, and allowed to dry [35,42,43]. Adekunle et al. report the synthesis of Co_3_O_4_ nanoparticles using CoCl_2_·6H_2_O and ammonium hydroxide as precursors and a precipitation and calcination method. Spherical-shaped nanoparticles sized 32.66 mm were obtained. In this chemical solution precipitation method, known concentrations of the precursors were allowed to react in the solution, after which the precipitate formed is then washed and dried [36]. The chemical solution precipitation method is of advantage because it is favorable for the large-scale production of materials; however, the particle morphology is not well defined, and it is not a very rapid technique. For these reasons, in the same study, the microwave method was used. In the microwave method, a solution containing the precursors is irradiated with microwave energy, after which the product is purified and dried [36,44]. The advantages of this method are that it is fast and highly reproducible, and there is better control over the quality and size of the nanoparticles produced [36]. In Adekunle et al.’s report, the Co_3_O_4_ nanoparticles were synthesized in a microwave method using CoCl_2_·6H_2_O and sodium hydroxide as precursors. Spherical-shaped nanoparticles sized 72.43 mm were obtained [36]. In another study, Co_3_O_4_ nanoparticles were synthesized using cobalt (II) nitrate hexahydrate and aqeuous ammonium hydroxide solution via microwave and calcination [39]. This yielded spherical agglomerated crystalline nanoparticles of about 13 nm in size. The microemulsion quenching technique has also been used in synthesizing Co_3_O_4_ nanoparticles due to the advantages of controlling the size, stability, and consistency of the nanoparticles [38]. This technique utilizes microemulsions containing desirable precursors, which produces a precipitate that is processed further by washing and drying [38,45,46,47]. Dogra et al. used cobalt-based metallosurfactants (i.e., bishexadecylamine cobalt dichloride, bishexadecyltrimethyl ammonium cobalt tetrachloride, and bisdodecylamine cobalt dichloride), and spherical nanoparticles ranging in size from 1–5 nm were obtained for all precursors [38].

### 2.2. Biological Methods

Many physicochemical methods may be disadvantageous because they are time-consuming, use toxic chemicals or high energy, or are otherwise environmentally unfriendly. There has been a push to use “green” synthesis methods [2,7] (Table 1), which are becoming increasingly popular. Proponents of bio-synthesis or green synthesis methods argue that the bio-synthesis of nanoparticles is easier, eco-friendlier, less time-consuming, more cost-effective and non-hazardous, and more advantageous in terms of operational cost and equipment exhibit efficiency [2,5]. This argument is open for debate and is not within the scope of this review. However, we discuss some of these synthesis methods as alternatives to physicochemical methods. Green synthesis methods include the use of some materials that occur naturally and are readily available. Such biosynthesis methods have used biological materials such as leaf extracts of Populus ciliate [7], extracts of other plant parts (the roots and fruits of several plant species for Co_3_O_4_ nanoparticle synthesis) [2], and microbes (such as bacteria, fungi, and yeast) as templates [2]. Similarly, as in the physicochemical synthesis methods, Co_3_O_4_ nanoparticles of different sizes, morphologies, or other unique properties were produced, depending on the biological materials and methods used [2]. The disadvantages of using biological methods for synthesis are not discussed quite as much compared to their advantages. It should be noted that with biological methods, challenges such as seasonal/climate variation in the concentrations of active biomolecules of microbes and plants may be a factor for their utilization in synthesis, since these biomolecules/phytochemicals act as reducing agents in synthesis [48]. These biomolecules may not be ideal as reducing agents for the synthesis of some nanoparticles that require strong reducing agents [48].

#### 2.2.1. Bio-Synthesis Using Plant Extracts

In what may be described as a bio-mediated method, Co_3_O_4_ nanoparticles were synthesized via a hot plate combustion method using cobalt (II) nitrate (Co(NO_3_)_2_·6H_2_O) as the oxidizing agent and glycine (C_2_H_5_NO_2_) and Punica granatum (pomegranate) seed extract as reducing agents [1]. This produced quasi-spherical-shaped, highly agglomerated nanoparticles of size ranging between 1–7 nm [1]. In another study, *Populus ciliata* leaf extract was used as a reducing agent, using cobalt nitrate hexahydrate (Co(NO_3_)_2_·6H_2_O)as the precursor for the synthesis of Co_3_O_4_ [7]. Well dispersed and uniform nanoparticles ranging between sizes 25–35 nm were the result [7]. Magdalane et al., 2019 synthesized Co_3_O_4_ nanoparticles using *Aspalathus linearis* leaf extract and cobalt nitrate (Co(NO_3_)_2_) via a hydrothermal method to obtain nanoparticles of irregular morphology, irregular shape and a size, ranging from 20–40 nm [12]. Using a plant extract, too, Dubey et al. report Co_3_O_4_ nanoparticles synthesized from cobalt acetate tetrahydrate (Co(CH_3_COO)_2_·4H_2_O, ammonia solution (NH_3_·H_2_O), and the latex of *Calotropis procera* [49].

#### 2.2.2. Bio-Synthesis Using Microbes

In addition to the plant extract-mediated synthesis of Co_3_O_4_ nanoparticles, microbe-mediated synthesis is also becoming popular. Omran et al., 2019 discuss the myco-synthesis of Co_3_O_4_ nanoparticles using the fungus *Aspergillus brasiliensis* ATCC 16404 and cobalt sulphate heptahydrate (CoSO_4_·7H_2_O) [3]. Parameters such as time, shaking speed, illumination, the dry weight of *Aspergillus brasiliensis* ATCC 16404, and concentrations of CoSO_4_·7H_2_O had to be optimized for the optimal yield of the Co_3_O_4_ nanoparticles [3]. Monodispersed, quasi-spherical-shaped Co_3_O_4_ nanoparticles of size 20–27 nm were obtained [3].

#### 2.2.3. Characterization Methods

**Figure 2:**
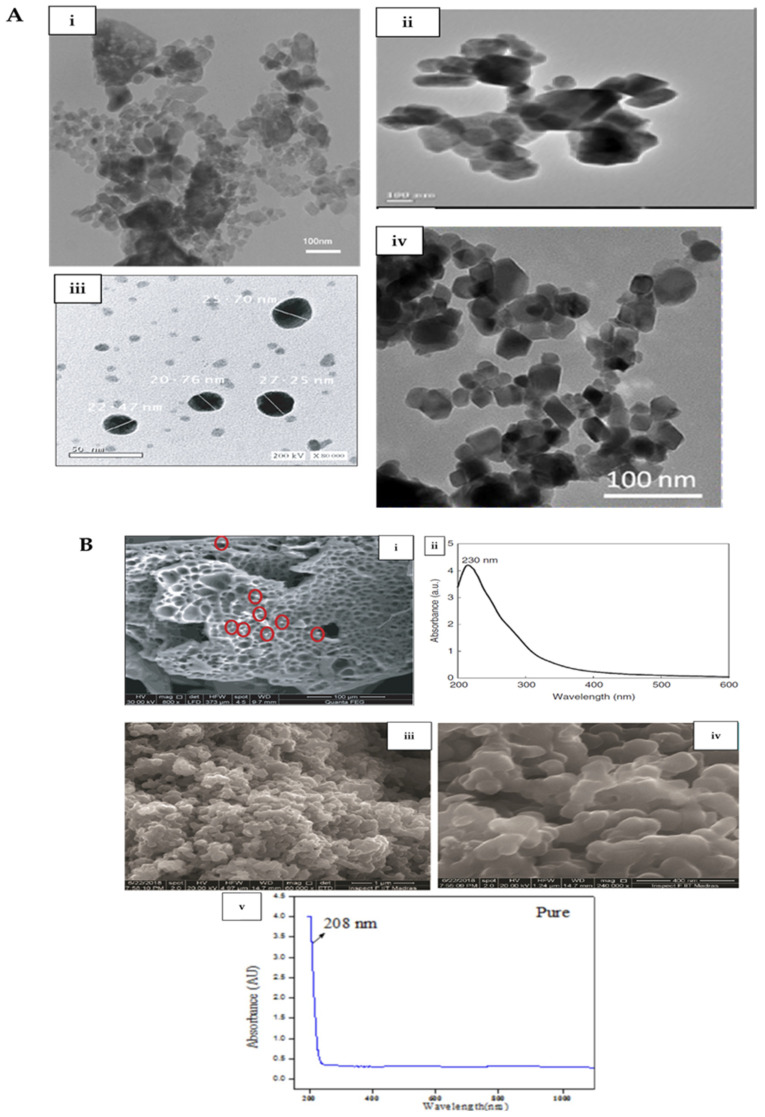
Electron microscopy and UV-Vis images of select nanoparticles. (**A**) Range of sizes of select Co_3_O_4_ nanoparticles and their nanocomposites: (i) transmission electron microscope micrograph of Co/Co_3_O_4_ nanocomposites synthesized according to the sonochemical method (∼20 nm in size). This image was adapted from Yousefi et al. [51] with permission from Elsevier. (ii) Transmission electron microscope micrograph of α-Fe_2_O_3_/Co_3_O_4_ nanocomposites synthesized via the co-precipitation method (average crystallite size of 25.34 nm). This image was adapted from Bhushan et al. [50] under the open access Creative Common CC BY license of Springer Nature. (iii) High-resolution transmission electron microscope image of myco-synthesized Co_3_O_4_ nanoparticles (20–27 nm in size). This figure was adapted from Omran et al. [3] with permission from John Wiley and Sons. (iv) Transmission electron microscope image of plant extract-synthesized Co_3_O_4_ nanoparticles (size 15–35 nm). This image was adapted from Hafeez et al. [7] under the open access Creative Common CC BY license of IOPScience. (**B**) Morphology and spectra of select Co_3_O_4_ nanoparticles used in antibacterial applications: (i) field scanning electron microscope micrograph of myco-synthesized Co_3_O_4_ nanoparticles showing their spherical-like morphology. (ii) UV/Vis absorption spectrum of myco-synthesized Co_3_O_4_ nanoparticles showing a distinct absorption peak at λ_280nm_. These images were adapted from Omran et al. [3] with permission from John Wiley and Sons. (iii,iv) High-resolution scanning electron microscope micrographs of spherical, agglomerated Co_3_O_4_ nanoparticles. (v) UV/Vis/NIR absorption spectrum of Co_3_O_4_ nanoparticles showing an absorption peak at λ_208nm_. These images were adapted from Mayakannan et al. [39] with permission from Elsevier.

Several methods have been used to characterize Co_3_O_4_ nanoparticles (Table 1, Figure 2) and its nanocomposites (Table 2, Figure 2) after synthesis. These can be broadly split into three main groups, namely microscopic methods, spectroscopic methods, and physicochemical property determination methods. These will be discussed briefly.

Microscopic methods include TEM, SEM, HRTEM, HRSEM, and FESEM. These microscopic techniques are useful for the shape, size, morphology, and micro-imaging determinations of the nanoparticles. The presence or absence of the aggregation of the nanoparticles can also be observed using these methods. Some of these microscopes may also be equipped with other characterization instrumentation, such as energy-dispersive X-ray spectroscopy, to provide information about the elemental composition of the nanoparticle being interrogated. This kind of information is important in interpreting antibacterial effects of the nanoparticles or nanocomposites and deducing why such effects are observed. For example, the antibacterial potential of a nanoparticle is determined by factors such as its size, morphology, and specific surface area [1].

Spectroscopic methods documented for the characterization of Co_3_O_4_ nanoparticles used for antibacterial studies include Fourier-transform infrared spectroscopy (FTIR), ultraviolet-visible spectroscopy (UV-Vis), X-ray photoelectron spectroscopy (XPS), energy-dispersive x-ray spectroscopy (EDS or EDX), X-ray diffraction spectroscopy (XRD), Raman spectroscopy, and photoluminescence spectroscopy (PL). The characterization of Co_3_O_4_ nanoparticles is performed using these methods to extract information about the crystallinity (XRD), raman active vibration modes (Raman), the elemental composition (EDS), energy band gap and spectra determination (UV-Vis), exciton and defect characteristics (PL), chemical bonding characteristics, and functional group determination (FTIR) of the nanoparticles. In the case of FTIR, for instance, such characteristics were used to gain a better understanding of the role of interactions between cobalt ions and bioactive molecules of mycelial cell-free filtrate in the mycosynthesis and stabilization of Co_3_O_4_ nanoparticles [3]. The characteristic morphology and spectra of Co_3_O_4_ nanoparticles used in antibacterial applications are shown in Figure 2. Physico-chemical property determination methods documented for the characterization of Co_3_O_4_ nanoparticles used for antibacterial studies include vibrating sample magnetometry (VSM) and dynamic light scattering (DLS). These methods provide information about the ionic charge, stability and average size determination (DLS), and magnetic properties (VSM) of the nanoparticles. Observed magnetic properties are important if considering the possibility of recycling nanoparticles via an external magnetic field for as many times as possible during wastewater remediation of the targeted pollutants.

## 3. Antibacterial Resistance and Antibacterial Activity

### 3.1. Antibacterial Resistance

**Figure 3:**
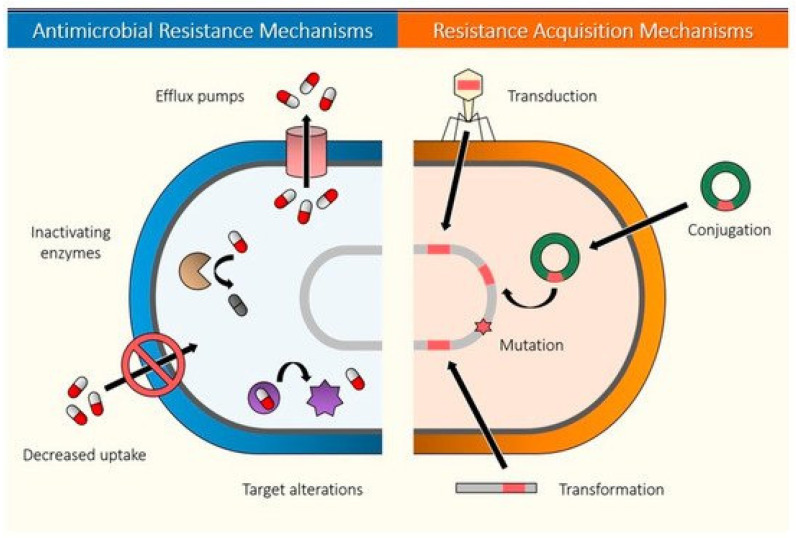
Mechanism of antibiotic resistance in antibiotic-resistant bacteria. Antibiotic-resistant bacteria may neutralize antibiotics via different mechanisms such as inactivating enzymes, altering their permeability to reduce antibiotics taken into the cell, eliminating antibiotics that enter the cell using efflux pumps, decreasing uptake of antibiotics, and modifying the targets of the antibiotics. Bacteria may acquire these resistance mechanisms via transduction, conjugation, mutation, and transformation. Reprinted from Álvarez-Martínez et al. [61] under the open access Creative Common CC BY license of MDPI.

Antibiotic resistance is a formidable global environmental and health threat. Historically, antibiotic-producing microbes were used to prevent and treat diseases more than 2000 years ago [16]. However, in modern times, the health threat of antibiotic resistance was recognized not too long after the discovery of antibiotics. For instance, salvarsan, the first synthetic antibiotic, was first used clinically in 1910 [16,54], followed by reported resistance in peer-reviewed work as early as 1924 [54,55,56]. Similarly, the discovery in 1928 by Alexander Fleming of penicillin, an antibiotic of natural origin, and of its clinical use in the 1940s, was quickly followed by resistance also in the 1940s [16,17]. Hence, the golden age of antibiotic discovery (1940–1960) [16,57] was rapidly followed by the “lean years” [57], when antimicrobial resistance had begun to erode the efficacy of antibiotics already in use, and the discovery of new antibiotics [16,57]. The end of the golden era of antibiotic discovery could be attributed to the increased use, misuse, and overuse of antibiotics for clinical and animal production [16,17,18,19], global migration, and selection pressure in the environment [17]. Mechanisms of antibiotic resistance (Figure 3) include the production of enzymes (e.g., β-lactamases) to effectively neutralize antibiotics possessing the β-lactam ring [17], the production of efflux pumps [17,58], the modification or breakdown of the antibiotic [17], cell wall adaptations [59], and the modification of the target of the antibiotic [59,60].

The environment is also now more steadily being recognized for its role in the spread of antibacterial resistance [20]. For example, a search in Web of Science using the keywords “antibiotic resistance”, and then within that search for “environment”, showed that publications on these topics with a sizable number of publications (68 publications) could be observed in 1997, whereas more than 100 publications were first observed in the year 2000. Since then, publications in this area have continued to increase at over 1000 publications per year since 2018. While it is surprising that the environment is only recently receiving attention for its role in sustaining the spread of antibacterial resistance, it is a welcome development. Both environmental and health threats are inseparable, as there is an inevitable intersection between the environment and health, such that anything that alters the quality of the environment also alters the quality of health. This relationship has also been communicated by the United Nations Environment Program (UNEP), which describes antimicrobial resistance as one of the six “emerging issues of environmental concern” in their “Frontiers 2017” report [62]. Similarly, the World Health Organization (WHO), in its report titled “global action plan on antimicrobial resistance”, recognizes that drug-resistant bacteria are present in food, water, and the environment [20,63]. Therefore, one of the proposed actions to achieve the fourth strategy of the “global action plan on antimicrobial resistance” is focused on addressing the presence of antimicrobial agents in the environment, with an emphasis on food, water, and wastewater [20]. Research investigating antibiotic resistance has found that antibiotic-resistant bacteria and antibiotic-resistant genes occur in environmental samples, including wastewater, which serves as an inadvertent reservoir of antibiotics, antibiotic-resistant bacteria, and antibiotic-resistant genes that are generated from agricultural, health, and other human activities [21]. The current methods of wastewater treatment do not completely eliminate antibiotics, antibiotic-resistant bacteria, and antibiotic-resistant genes in wastewater [21,22], thus requiring the use of other novel methods in addition to current wastewater treatment strategies. In the traditional wastewater treatment system, wastewater is treated using the pre-primary, primary, secondary, and tertiary treatment stages [22,64,65]. Specific processes during these treatment stages have varying degrees of efficiency in eliminating antibiotic-resistant bacteria and genes [25,66]. For example, anaerobic and aerobic treatment reactors are not efficient in removing antibiotic-resistant bacteria and genes when used individually [25,66]. Concerns in using other wastewater treatment strategies include the persistence of antibiotic resistance genes after the elimination of antibiotic-resistant bacteria via advanced oxidation processes, and constructed wetlands [25,66]. The presence of these antibiotic-resistant genes even after the elimination of bacteria is concerning because of the risk of the continued spread of antibiotic resistance via horizontal gene transfer. Allowing antibiotic-resistant bacteria and genes to persist after treatment in municipal wastewater treatment plants and after discharge contributes to the continued incidence of antibiotic resistance in society. This is because these antibiotic-resistant bacteria genes are then mobilized into surrounding waters, sediments, and soil [66]. Some studies have shown that some wastewater plants have higher concentrations of antibiotic-resistant genes than surrounding waters, sediments, and soil, though they have identical antibiotic-resistant genes as the wastewater plants [66]. This suggests that wastewater contaminated with antibiotic-resistant bacteria and antibiotic-resistant genes is a source of this pollution in the environment. Chlorination is more effective than the other commonly used disinfection methods (ultraviolet irradiation and ozone) against antibiotic-resistant bacteria and genes [25,66]. However, it contributes to antibiotic resistance via natural transformation [67,68].

Alternative methods are therefore needed as additional steps in effective wastewater treatment with respect to antibiotic resistance. One approach to doing this, as shown in the literature, is via the use of nanoparticles. In general, nanoparticles have been researched for wastewater treatment for several pollutants [23,24,69,70]. Additionally, the use of nanoparticles to combat antibiotic resistance via the remediation of contaminated wastewater has been suggested and is of recent research interest.

### 3.2. Nanoparticles and Wastewater Remediation

The sustainable availability of clean water remains a global challenge due to problems such as pollution exacerbated by anthropogenic activities, the increasing world population, and emerging contaminants [23]. Nanotechnology is one of the additional technologies that have been investigated on the laboratory scale, pilot scale and in-situ [23] for use with current wastewater treatment technologies. Promising results have also been observed using nanoparticles to purify water from seawater [71], an approach needed to supplement the scarce freshwater resources that are primary sources of clean water. Nanoparticles such as carbon-based nanoparticles, metal nanoparticles, magnetic nanoparticles, transition metal sulfide nanoparticles, silica-based nanomaterials, organic polymer nanomaterials, biogenic nanoparticles, and metal oxide nanoparticles have been used for the remediation of pollutants such as dyes, antibiotics, other pharmaceutical compounds, heavy metals, organic compounds (e.g., phenolic-based compounds, benzene-based compounds, hydrocarbons), and microbes [23,69,72,73], depending on the affinity of each nanoparticle for the contaminants.

#### 3.2.1. Nanoparticles and Remediation of Bacteria

**Figure 4:**
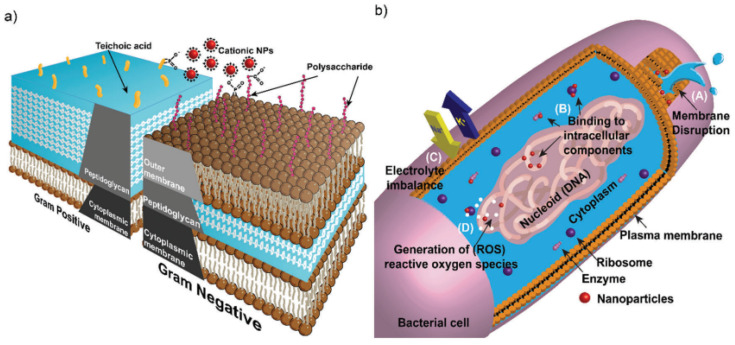
Graphic representation showing: (**a**) cell wall structures of gram-positive and gram-negative bacteria. (**b**) Antibacterial mechanism of nanoparticles. (A) Cell membrane is disrupted, leading to cell leakage. (B) Nanoparticles can bind to cellular components. (C) Electron transport is disrupted, thereby leading to electrolyte imbalance. (D) Reactive oxygen species are generated. Reproduced from Gupta et al. [74] with permission from the Royal Society of Chemistry.

As discussed previously, the potential of nanoparticles for the remediation of wastewater laden with antibiotic-resistant bacteria and genes is being widely studied. Harmful by-products of chemical disinfection of wastewater treatment [23] are another reason that nanoparticles are being proposed for such remediation. Nanoparticles may exhibit biocidal activity against bacteria, act as disinfectants in water when activated with light, or be used as antifouling agents in wastewater treatment [71]. While the mechanisms of such remediating activity of nanoparticles on bacteria is not well understood, some insight from research has been provided (Figure 4).

Nanoparticles may disrupt bacterial membranes and hinder biofilm formation [26,27]. The latter is important because the disruption of biofilms helps prevent bacterial resistance, since biofilms “shield” multiple microbes and serve as a hotbed for resistance mutations to develop [26]. The former is also important because contact with the bacterial cell is crucial before any remediating activity on the bacteria is possible [26]. Such contact is made possible through interactions such as hydrophobic interactions, van der Waals forces, receptor–ligand interactions, and electrostatic attraction [26,74]. Any damage to the cell wall or cell membrane make bacteria more vulnerable to the external environment [26]. After membrane disruption, interactions of the nanoparticles with the cellular contents of bacteria may further inflict damage on the bacteria. Such cellular components and targets include DNA, ribosomes, enzymes, other proteins, lysosomes, alterations in electrolyte balance, alterations in gene expression, and oxidative stress [26].

#### 3.2.2. Metal Oxide Nanoparticles and Remediation of Bacteria

**Figure 5:**
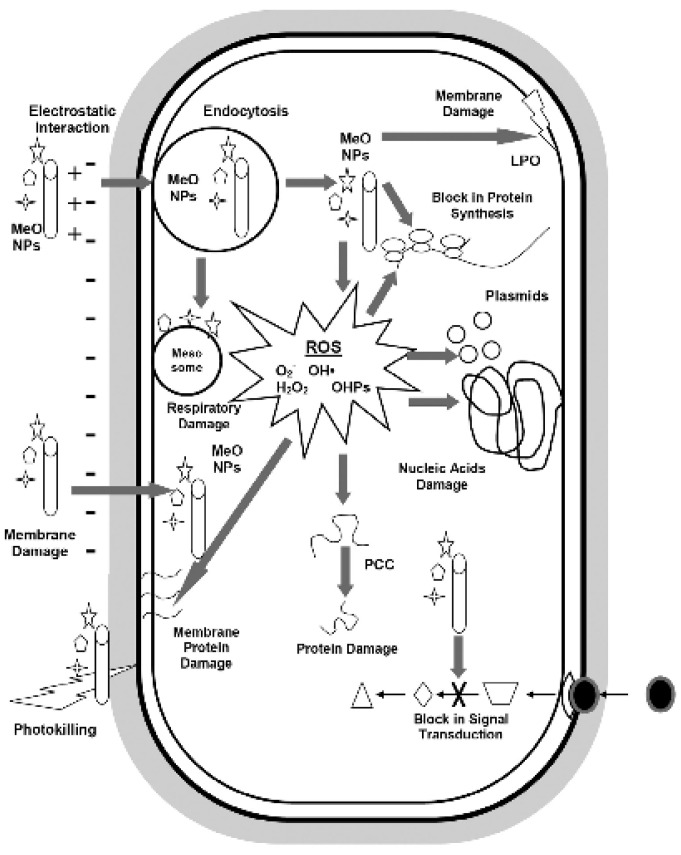
Graphic representation showing the antimicrobial mechanisms of metal oxide nanoparticles. Reproduced from Raghunath and Perumal [44] with permission from Elsevier.

Metal oxides are also known to exhibit antibacterial or inhibitory effects on different bacteria via various mechanisms (Figure 5) [27].

They are inherently photocatalytic, which leads to generation of generation of reactive oxygen species (ROS) [27], and they primarily exhibit antimicrobial activity via this mechanism [44]. For example, iron oxide nanoparticles damage the intracellular content of bacteria primarily due to the generation of reactive oxygen species (ROS) [27]. Zinc oxide (ZnO) and copper oxide (CuO) nanoparticles also inflict similar damage to bacteria via the generation of ROS, and they also inhibit biofilm development [27]. CuO nanoparticles may in addition interfere with the nitrogen metabolism of the bacteria cell [26]. ROS generation is also a factor in bacteria cell damage with other nanoparticles such as aluminum oxide (Al_2_O_3_) and titanium oxide (TiO_2_) [26]. TiO_2_ nanoparticles may in addition induce compression, degeneration, and fragmentation of DNA [26].

Not all antibacterial or inhibitory activity can be attributed to ROS generation. Dissolved metal ions that are slowly released from metal oxides also play a role by interacting with the functional groups of nucleic acids and proteins, modifying enzyme activity and inducing other alterations to the normal functioning of the cell [26]. However, this is a less important antibacterial mechanism of the metal oxide nanoparticles when compared with ROS generation [26]. Other less defined antibacterial mechanisms of metal oxides exist and are non-oxidative mechanisms [26]. For example, magnesium oxide (MgO) nanoparticles have antibacterial effects, yet analyses show a lack of ROS generation, lack of lipid peroxidation, or significant presence of nanoparticles in the bacteria cell; however, other metabolic processes of the bacteria are noticeably affected [26]. Other mechanisms of metal oxide nanoparticles on bacteria (Figure 5) include cell membrane damage via electrostatic interaction, photokilling, disruption in metal/metal ion homeostasis, genotoxicity, and alteration of protein and enzyme function [44]. In the next section, we discuss the findings on the antibacterial activity of cobalt oxide (Co_3_O_4_) nanoparticles and their applications for the antibacterial environmental remediation of wastewater.

### 3.3. Cobalt Oxide-Based Nanoparticles and Their Antibacterial Applications

#### 3.3.1. Antibacterial Activity of Cobalt Oxide Nanoparticles and Mechanisms

Co_3_O_4_ nanoparticles have unique structural, chemical, physical, magnetic, and optical properties, making them useful for several applications [75]. They have hence been used in a wide range of applications, including the manufacture of materials such as lithium ion batteries, capacitors, gas sensors, field emission materials, magneto-resistive devices, energy storage systems, and they are also used for catalysis [7]. The properties of Co_3_O_4_ nanoparticles have been taken advantage of in environmental remediation applications, such as the degradation of dyes, dye waste, and antibiotics, similar to some applications of other nanoparticles. These include the photocatalytic degradation of hazardous dye waste in wastewater using Co_3_O_4_ nanostructures synthesized with *A. linearis* leaf extract [12], the photocatalytic degradation of hazardous dye waste and the catalytic reduction of 4-nitroaniline and 4-nitrophenol using Co_3_O_4_ nanoparticles synthesized with *Azadirachta indica* leaf extract [76], and methyl orange dye adsorption using Co_3_O_4_ nanoparticles [77].

**Figure 6:**
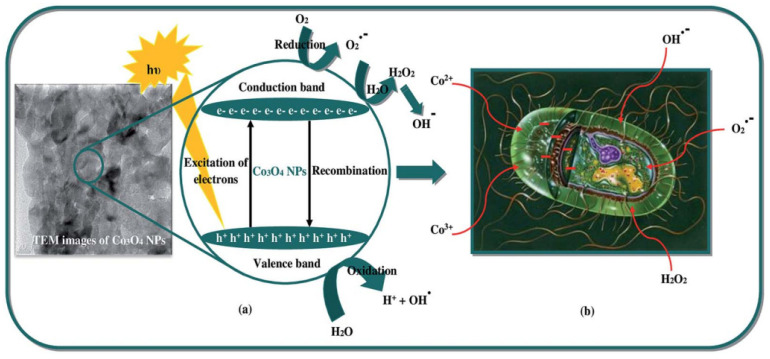
Graphic representation showing the antibacterial mechanisms of cobalt oxide nanoparticles: (**a**) formation of OH after light irradiation of nanoparticles. (**b**) Inhibition of bacterial growth after exposure to nanoparticles. Reproduced from Sivachidambaram et al. [76] with permission from the Royal Society of Chemistry under the open access Creative Common CC BY license of RSC Advances.

The antibacterial effects of Co_3_O_4_ nanoparticles are also documented in the literature (Table 3). Co_3_O_4_ nanoparticles were shown to exhibit antibacterial activity towards *Staphylococcus aureus*, *Bacillus subtilis*, *Escherichia coli,* and *Pseudomonas aeruginosa* in a work by Jesudoss et al., 2017. The authors suggest that the antibacterial properties of Co_3_O_4_ nanoparticles are dependent on properties such as size, morphology, and specific surface area, although they consider the exact mechanism of antibacterial activity to be vague [1]. The electrostatic attraction and formation of reactive oxygen species (Figure 6) are proposed as possible mechanisms of antibacterial activity based on previously reported research. Jesudoss et al., however, propose other additional possible mechanisms. In one approach, the positive oxidation states of Co_3_O_4_ nanoparticles can interact with negatively charged portions of the bacterial cell, hence inducing destruction of the cells [1]. In another approach, electron-hole pairs are formed after irradiation of the spinel-structured Co_3_O_4_ nanoparticles such that the excited electrons react with oxygen molecules, leading to the formation of superoxide radical ions succeeded by the production of hydrogen peroxide. At the same time, the holes induce the production of hydroxyl radicals when they react with water. Both superoxide radical ions and hydroxyl radicals that come in contact with the cell wall of the bacteria disintegrate its proteins and lipids [1]. In a similar study, Co_3_O_4_ nanoparticles were found to inhibit the growth of gram-positive bacteria (*Bacillus subtilis* and *Staphylococcus aureus*) and gram-negative bacteria (*Pseudomonas aeruginosa* and *Escherichia coli*) [76]. Using the disk diffusion method, the Co_3_O_4_ nanoparticles inhibited the growth of all bacteria compared to a chloramphenicol standard. The zone of inhibition varied depending on the tolerance of each bacteria. The Co_3_O_4_ nanoparticles were more inhibitory than the antibiotic standard except for in *E. coli* [76]. These two research efforts were aimed at showing the efficacy of Co_3_O_4_ nanoparticles for the photocatalytic degradation of dye in wastewater and antibacterial activity for biomedical applications. However, the activity of Co_3_O_4_ nanoparticles towards both dye effluents and bacteria demonstrates their potential for the simultaneous remediation of these contaminants in wastewater that contains diverse pollutants.

The antimicrobial studies of Omran et al., 2019 showed that mycosynthesized Co_3_O_4_ nanoparticles were effective against both gram-positive bacteria (*Bacillus subtilis* and *Staphylococcus aureus*) and gram-negative bacteria (*Pseudomonas aeruginosa* and *Escherichia coli*), but ineffective against *Candida albicans*. The antibacterial effects of the Co_3_O_4_ nanoparticles were similar to the antibacterial effects of the antibiotics (streptomycin, ampicillin, gentamycin and erythromycin) used for comparison in this study [3]. The authors note the importance of these effects in the potential of the Co_3_O_4_ nanoparticles for wastewater treatment and water disinfection. More so, the magnetic properties of the nanoparticle, if used for wastewater treatment, are an added advantage, presumably for recycling the nanoparticles.

Co_3_O_4_ nanoparticles synthesized by Hafeez et al., 2020 also demonstrated antibacterial effects against both gram-positive bacteria (*Bacillus subtilis* and *Bacillus lichenformis*) and gram-negative bacteria (*Klebsiella pneumoniae* and *Escherichia coli*), with better inhibitory effects with the nanoparticles than when an antibiotic (bacitracin) was used for comparison [7]. Antibacterial effects were also more effective when used in the gram-positive bacteria and less effective in the gram-negative bacteria. From a mechanistic interpretation, this was attributed to the differences between the cell walls of both types of bacteria. The cell wall of gram-positive bacteria is more porous and permeable than the cell walls of gram-negative bacteria [7]. After penetration of the cell wall, damage may also be inflicted on the cell membrane, DNA, and proteins by the formation of reactive oxygen species (ROS), for example hydrogen peroxide, that are formed in the presence of metallic ions [7]. While the authors of this work do not explicitly conduct this research with a wastewater treatment application in mind, the results demonstrate the potential use of their Co_3_O_4_ nanoparticles for wastewater treatment.

While a common theme for explaining the antibacterial activity of Co_3_O_4_ nanoparticles to bacteria that occurs across research are the size effects of nanoparticles, this mechanism is not quite clear and has not been elaborately investigated or discussed. It has, however, been suggested generally for nanoparticles that decreasing sizes of nanoparticles are particularly favorable with respect to the increased surface area of nanoparticles interacting with bacteria [25,78,79,80]. Other proposed reasons related to smaller size effects include ease of penetration of electrons, improved adhesion, absorption, and interaction with the bacteria cell, after which the nanoparticles enter the cell to inflict damage [3,7,25,76]. The shape of the nanoparticles also enhances the contact killing of microbes through improved forces for local adhesion [25].

Antibiotic resistance is a global threat, hence positioning nanoparticles as alternatives to antibiotics for antibacterial applications. Ironically, bacteria are also known to develop resistance to some nanoparticles and/or induce resistance to certain antibiotics. Examples include bacterial resistance to silver nanoparticles [81], facilitation of horizontal gene transfer of antibiotic resistant genes by silver nanoparticles [82], adaptive bacteria resistance to zinc oxide (ZnO) nanoparticles (which are unstable and revert to sensitivity after a number of days of in the absence of the nanoparticles) [83], facilitation of horizontal gene transfer of antibiotic resistant genes by ZnO nanoparticles [84], aluminium oxide (Al_2_O_3_) enhancement of the conjugative transfer of antibiotic resistance genes in the environment [85], and the induction of soil microbial resistance to tetracycline via co-selection and horizontal gene transfer on exposure to rare earth oxide nanoparticles such as lanthanum (III) oxide (La_2_O_3_), neodymium (III) oxide (Nd_2_O_3_), and gadolinium oxide (Gd_2_O_3_) [86]. Hence, nanocomposites have been proposed as alternatives to “single component” nanoparticles. The rationale behind this is that multiple layers of different nanoparticles create several hurdles for bacteria to overcome in order to be resistant to the nanocomposite. Subsequently, we discuss examples of the antibacterial effects of such nanocomposites containing Co_3_O_4_ or nanoparticles decorated with Co_3_O_4_.

#### 3.3.2. Antibacterial Activity of Cobalt Oxide-Based Nanocomposites and Mechanisms

Environmental remediation of cobalt oxide-based nanocomposites or doped cobalt oxide nanoparticles includes the use of Co_3_O_4_ nanoparticles as well as Co_3_O_4_ nanoparticles doped with NiO and PdO/Pd as nano-catalysts to degrade methyl orange in the presence of sunlight [13], the photodegradation of crystal violet dye via Helianthus annuus leaf extract-synthesized Co_3_O_4_ nanoparticles and Ag-Co_3_O_4_ heterostructures [14], and the use of agar-immobilized Co_3_O_4_ nanoparticles for the catalytic reduction of congo red, methyl blue, and 4-nitrophenol in the presence of sodium borohydride [15]. Xu et al. demonstrated the use of the degradation of chloramphenicol using a biochar-supported Co_3_O_4_ nanocomposite via peroxymonosulfate activation [87].

Some research has also investigated the antibacterial potential of Co_3_O_4_-based nanoparticles (Table 4). In one study, the potential of Au-graphene oxide-Co_3_O_4_ hollow spheres for binding antibiotic-resistant genes was investigated by Yu et al., where a strong interaction between cobalt or Co_3_O_4_ with deoxyribonucleic acid (DNA) enhances its capability to bind to DNA [86] for deactivation. Inhibition of the genetic replication of the antibiotic-resistant genes by the hollow spheres was observed, which was proposed to occur via the intercalation mechanism of the Co_3_O_4_ component and a groove binding mechanism of the entire hollow spheres [88]. The importance of this finding for the purification of antibiotic-resistant genes in contaminated water was highlighted in this work. In another study, the antibacterial activities of a β-CoMoO_4_-Co_3_O_4_ nanocomposite were demonstrated against *Escherichia coli*, *Pseudomonas aeruginosa,* and *Staphylococcus aureus,* probably due to electrostatic attraction and the formation of reactive oxygen species [89]. Bhushan et al. also investigated the antibacterial effects of a novel nanocomposite consisting of α-Fe_2_O_3_ and Co_3_O_4_. The α-Fe_2_O_3_/Co_3_O_4_ nanocomposite was synthesized using the co-precipitation method. During the reaction, the concentration of the precursor for hematite (α-Fe_2_O_3_) was kept constant, and the concentration of the precursor for the α-Co_3_O_4_ nanoparticle varied in increments to obtain four different α-Fe_2_O_3_/Co_3_O_4_ nanocomposites. The antibacterial properties of these four α-Fe_2_O_3_/Co_3_O_4_ nanocomposites and the pure α-Fe_2_O_3_ and Co_3_O_4_ nanoparticles were investigated on *Bacillus subtilis* and *Staphylococcus aureus* (gram-positive bacteria) and on *E. coli* and *Salmonella typhi* (gram-negative bacteria). Based on the zone of inhibition data obtained via the Bauer–Kirby disc diffusion method, the α-Fe_2_O_3_/Co_3_O_4_ nanocomposites with the highest concentrations of cobalt were the most effective against the tested bacteria, particularly *E. coli* and *S. aureus* [50]. In experiments using α-Fe_2_O_3_ and Co_3_O_4_ nanoparticles separately via the Bauer–Kirby disc diffusion method, α-Fe_2_O_3_ was more effective in inhibiting bacteria growth [50]. Growth experiments of the bacteria in α-Fe_2_O_3_/Co_3_O_4_ nanocomposites and in the pure α-Fe_2_O_3_ and Co_3_O_4_ nanoparticles were also investigated. Bacteria were grown in growth media and increasing concentrations (45, 60, 75, 90 and 120 mg/dL) of Fe_2_O_3_/Co_3_O_4_ nanocomposites, and in the pure α-Fe_2_O_3_ and Co_3_O_4_ nanoparticles in separate experiments. Expectedly, bacteria in the growth media thrived, and bacteria in the treatments of Fe_2_O_3_/Co_3_O_4_ nanocomposites and in the α-Fe_2_O_3_ and Co_3_O_4_ nanoparticles grew less favorably and with a general trend of less growth as concentration increased [50]. Bacteria grew comparatively in α-Fe_2_O_3_ and Co_3_O_4_ nanoparticles, with slightly increased antibacterial effects in α-Fe_2_O_3_ [50]. Generally, the Fe_2_O_3_/Co_3_O_4_ nanocomposites exhibited superior bactericidal effect compared to the pure α-Fe_2_O_3_ and Co_3_O_4_ nanoparticles. This could be attributed to the synergistic effect of both α-Fe_2_O_3_ and Co_3_O_4_ nanoparticles, which individually possess antibacterial properties and may increase the generated oxygen free radicals by the nanocomposites on interaction with the bacteria [50]. Interestingly, the Fe_2_O_3_/Co_3_O_4_ nanocomposites with equimolar concentrations of both α-Fe_2_O_3_ and Co_3_O_4_ at 120 mg/dL concentration largely reduced the growth of *B. subtilis* and *S. typhi,* whereas the nanocomposite at the same concentration decimated *S. aureus* and *E. coli* growth [50]. These studies show the potential of these nanocomposites for bacteria wastewater remediation.

In another study, the application of a Co/Co_3_O_4_ nanocomposite was applied to the remediation of organic dye in wastewater and the antibacterial effects on *B. subtilis*, *S. aureus*, *P. aeruginosa*, *K. pneumonia*, and *E. coli*. Based on the MIC, the nanocomposite was moderately effective against all bacteria, where P. aeruginosa was most susceptible to the nanocomposite [51]. Mayakannan et al. also investigated the antibacterial activities of nickel-doped-Co_3_O_4_ nanoparticles. *B. subtilis*, *S. aureus*, *P. aeruginosa*, and *E. coli* were also used in this study. The growth of all bacteria was inhibited by pure and doped Co_3_O_4_ nanoparticles [39]. A core shell of Co_3_O_4_@ZrO_2_ was also used in a different study for wastewater and biomedical applications. The photocatalytic degradation of Rhodamine B dye and the antibacterial effect of the core/shell on *B. subtilis*, *S. aureus*, *P. aeruginosa*, and *E. coli* was studied. The effect of the core/shell was pronounced for *S. aureus* and *P. aeruginosa* [53]. From these studies, clearly some of these nanocomposites demonstrate the ability to remediate at least one pollutant other than antibiotic-resistant bacteria. This is promising, as the use of nanoparticles that can simultaneously remove multiple pollutants is desirable [90].

## 4. Environmental Impact

Nanoparticles are being used increasingly for several applications due to their unique properties. This exponential use of nanomaterials inadvertently leads to their mobilization into air, water, and soil [91], and subsequent interaction with different forms of life. An understanding of the interactions between different types of nanomaterials with the environment has only received attention for thorough study in the past two decades largely due to a better understanding of nanomaterials and the availability of analytical tools that make such inquiry and investigation possible [92]. Pathways by which nanomaterials specifically engineered for a purpose enter the environment are at the production phase, the consumer phase, or at disposal [93]. Therefore, ideal approaches to studying the interactions of synthetic nanomaterials with the environment are at all stages of the life cycle of the nanomaterial, including at the industrial stage, during public use, and the various stages and sinks involved in disposal. In addition, rigorous investigation of environmental interactions of nanoparticles at the research stage are equally ideal.

There is scarce but useful data that provide some insight into the environmental impact of Co_3_O_4_ nanoparticles. Here, we discuss some such studies. In one study, the toxicity of different nanoparticles including Co_3_O_4_ nanoparticles were investigated in zebra embryos and developing larvae. The authors found that the nanoparticles had differential effects depending on the developmental stage of the zebrafish, the type of the nanoparticle, and the size of the nanoparticle [94]. For example, nickel oxide (NiO) and chromium oxide (Cr_2_O_3_) nanoparticles were more toxic at the embryonic stage, whereas Co_3_O_4_ nanoparticles (of size 30 nm) were more toxic to the zebra fish embryo [94]. Additionally, Co_3_O_4_ nanoparticles (of size 30 nm) inflicted more damage to the skin of the zebrafish larvae than Co_3_O_4_ nanoparticles (of size 100 nm) [94]. Another study looked at the effect of Co_3_O_4_ nanoparticles on soil microbiota. The results of that research showed that Co_3_O_4_ nanoparticles have inhibitory effects associated with soil parameters connected to sulfur and phosphorus cycles [95]. Extracellular and intracellular effects of Co_3_O_4_ nanoparticles on marine algae manifesting in growth suppression, ROS generation, and waning chlorophyll *a* of algae have also been reported in the literature [96,97]. In another study, less impact on the environment was reported. For example, Dubey et al. describe an eco-toxic investigation involving the use of “green” synthesized Co_3_O_4_ nanoparticles [49]. Antibacterial assays using the Kirby–Bauer disc diffusion technique indicated poor antibacterial activity in the understudied bacteria. High concentrations above 100 µg/disc of Co_3_O_4_ nanoparticles were needed to detect zones of inhibition ranging from 7–17 mm in *E. coli*, *Pseudomonas* sp., *Alcaligenes* sp. (all gram-negative bacteria), and *Enterococcus* sp. (gram-positive bacteria) compared to zones of inhibition ranging from 19–55 mm when using ampicillin of the same concentrations [49]. Therefore, the authors suggest that this poor antibacterial activity may make Co_3_O_4_ nanoparticles safer for other applications with reduced negative impact on the environment [49]. Despite the obvious advantages and potential of Co_3_O_4_ nanoparticles for bacteria remediation, its impact on unintended targets in the environment will also need further scientific research.

## 5. Future Directions and Outlook

Antibacterial mechanisms of Co_3_O_4_ nanoparticles that are reported are not always experimentally determined in each of these studies. Formation of ROS is the commonly accepted mechanism based simply on previous literature. Many reports propose a mechanism to explain the antibacterial effects of Co_3_O_4_ nanoparticles and its nanocomposites without supporting data. Therefore, many mechanistic interpretations are speculative, as it is not clear that the antibacterial mechanism of Co_3_O_4_ nanoparticles and its nanocomposites are universal regardless of precursors, synthesis methods, nanocomposite contents, bacteria strains, and conditions of nanoparticle–bacteria interactions. Just like the metal oxides, there may be different mechanistic approaches for antibacterial activities of Co_3_O_4_ nanoparticles. The antibacterial mechanisms of Co_3_O_4_ nanoparticles and its nanocomposites may not necessarily be completely identical to the mechanisms of other metal oxide nanoparticles or nanoparticles in general. Thus, knowledge in this area is relatively scant, therefore necessitating further research. Although current knowledge of antibacterial activity of Co_3_O_4_ nanoparticles and its nanocomposites is very useful, more knowledge and understanding into these mechanisms is important for easier adoption in real-life remediation applications.

With respect to reports of the toxicity of Co_3_O_4_ nanoparticles, finding data that show the toxicity of any nanoparticle, including Co_3_O_4_ nanoparticles, is helpful. This is because the absence of information about the toxicity of a substance is not indicative of its safety. Good science that confirms the safety of nanoparticles, is pertinent to help inform good policy. Furthermore, knowing what properties make a nanoparticle toxic will also help drive the design, engineering, and synthesis of nanoparticles tailored for desired targeted applications, while keeping the balance of their safety for the overall environment. Balance needs to be made with respect to “safety by design” of nanoparticles to obtain Co_3_O_4_ nanoparticles suited for the purpose they were designed for while simultaneously having low toxicity. Thorough elucidation of the safety of these nanoparticles is important. While this balance may be a difficult feat to achieve, an alternative is to use Co_3_O_4_ nanoparticles in controlled conditions. Controlled settings such as engineering controls at a wastewater plant or the immobilization of Co_3_O_4_ nanoparticles into a suitable polymer may reduce or eliminate their mobilization into the environment and negative impact on the environment. The adoption of Co_3_O_4_ nanoparticles and other nanoparticles for wastewater remediation of antibiotic-resistant and/or pathogenic bacteria may also hinge on factors such as cost effectiveness, efficiency in remediation, and feasibility of recovering and recycling the nanoparticles for wastewater remediation. Cautious optimism can be applied to the use of Co_3_O_4_ nanoparticles for these applications.

## 6. Conclusions

The use of Co_3_O_4_ nanoparticles for technological, biomedical, and environmental applications is well documented in the literature. There is increasing interest in the bacteria remediation potential of Co_3_O_4_ nanoparticles and its nanocomposites due to the contribution of wastewater to the prevalence of antibiotic resistance in the environment. Research has demonstrated the antibacterial or inhibitory effects of Co_3_O_4_ nanoparticles. Although a distinction between biostatic and biocidal effects of Co_3_O_4_ nanoparticles and their nanocomposites is not always made, their antagonistic effects on pathogens, antibiotic resistant bacteria, gram-negative bacteria, and gram-positive bacteria are clearly seen in the literature. Research in this area is, however, in its infancy and needs more attention. Specifically, up-to-date experimentation is the primary research approach, and antibacterial mechanisms of Co_3_O_4_ nanoparticles are not always determined by experimental observation. In addition, there are many physiochemical factors of the nanoparticles that may affect their interactions with bacteria and antibacterial activity and results. Hence, it is necessary to have better design and control of the synthesis of Co_3_O_4_ nanoparticles for desired size, morphology, structure, and the resultant physicochemical properties with respect to the antibacterial reaction and mechanism. Hence, it would be beneficial to incorporate computational tools for simulating and modeling the interactions of nanoparticles with bacteria for insightful understanding of the reaction mechanisms, and machine learning to handle large-scale data from both experimental and computational work to examine the impact of the physiochemical properties of the nanoparticles on the antibacterial effects and optimization.

## Figures and Tables

**Table 1:** Cobalt oxide nanoparticle synthesis, characterization, and characteristics.

Material	Synthesis Method	Characterization Method	Morphology	Size	Reference
Co_3_O_4_	Biological (plant extract) synthesis and hot plate combustion method	XRD, FTIR, Raman, HRTEM, EDS, and UV-Vis	Quasi-spherical shape and high agglomeration	1–7 nm	[1]
Co_3_O_4_	Biological (myco-) synthesis	DLS, EDS, FTIR, VSM, FESEM, HRTEM	Quasi-spherical shape and monodispersed	20–27 nm	[3]
Co_3_O_4_	Biological (plant extract) synthesis	TEM, SEM, XRD, FTIR	Square-shaped, and aggregated	15–35 nm	[7]
Co_3_O_4_	Biological molecule-based synthesis	FTIR, XRD, SEM, TGA	Mixture of octahedron, tetrahedron, spheroidal, flakelike morphologies	20 nm–2 µm	[40]
Co_3_O_4_	Biological molecule-based synthesis	AFM, XPS	Spherical	2.5–3 nm	[41]
Co_3_O_4_	Microwave synthesis and calcination	XRD, UV, FTIR, HRSEM, PL, TEM	Spherical and agglomerated	13 nm	[39]
Co_3_O_4_	Precipitation and calcination	FTIR, SEM, TEM, XRD, UV-Vis	Spherical, interconnected, layered structure	32.66 nm	[36]
Co_3_O_4_	Microwave synthesis and calcination	FTIR, SEM, TEM, XRD, UV-Vis	Spherical, interconnected, layered structure	72.43 nm	[36]
Co_3_O_4_	Casting technique and calcination	XRD, TEM, IR, UV-Vis	Cubic, no agglomeration	13 nm	[35]
Co_3_O_4_	Microemulsion quenching technique	TEM, FESEM, EDS, XRD, UV-Vis	Spherical	1–5 nm	[38]
Co_3_O_4_	Laser ablation	UV-Vis-NIR, TEM, SEM, XRD, FTIR, PL, DLS, VSM	Spherical with some agglomeration	10 nm	[33]
Co_3_O_4_	Laser ablation	TEM, Raman, UV-Vis, XPS, CV	Spherical, agglomerated	∼2.5 nm	[32]
Co_3_O_4_	Laser fragmentation	XRD, TEM, EDS, XPS, Raman, FTIR	Uniform, spherical, well dispersed	∼5.8 nm	[34]

**Table 2:** Synthesis, characterization, and characteristics of cobalt oxide nanocomposites.

Material	Synthesis Method	Characterization Method	Morphology	Size	Reference
α-Fe_2_O_3_-Co_3_O_4_	Co-precipitation and calcination	XRD, TEM, EDS, VSM, Raman	Mixture of rod-shaped and hexagonal	25.34 nm (crystallite size)	[50]
Ni doped-Co_3_O_4_	Microwave synthesis and annealing	XRD, UV-Vis-NIR, FTIR, HRSEM, TEM, Fluor, EDS	Nanocubes	15–41 nm	[39]
Co/Co_3_O_4_	Sonochemical method	SEM, FTIR, XRD, VSM, EDS, CV	Snowballs	∼20 nm	[51]
MnFe_2_O_4_-Co_3_O_4_	Sonochemical co-precipitation method	HRTEM, EDS, XRD, PL, DRS, VSM, FTIR	MnFe_2_O_4_ nanorods attached to Co_3_O_4_ nanostructures	Not indicated	[37]
polyhydroxybutyrate-Co_3_O_4_	Co-precipitation method	FTIR, UV-Vis, XRD, SEM, EDS, TEM, TGA, DTA	Uneven surfaced structure, agglomerated; well dispersed Co_3_O_4_ in biopolymer	Not indicated	[52]
Co_3_O_4_@ZrO_2_	Sol-gel method	UV-Vis, FTIR, CV, FESEM, XRD	Spherical with irregular morphology; agglomerated	378.8 nm and 681.4 nm	[53]

**Table 3:** Cobalt oxide nanoparticles and bacteria remediation capability.

Target Bacteria in Study	Method of Assessing Activity on Bacteria	Concentration Used	Contact Time and Other Conditions	Antibacterial/Inhibitory Activity	Summary of Mechanism of Antibacterial/Inhibitory Activity	Reference
*S. aureus*	Disc diffusion method	0.001 g/10 mL	Incubated at 37 °C for 24 h	18.6 mm zone of inhibition	Probably cell membrane disruption and oxidative stress from ROS	[1]
*B. subtilis*	Disc diffusion method	0.001 g/10 mL	Incubated at 37 °C for 24 h	20.8 mm zone of inhibition	Probably cell membrane disruption and oxidative stress from ROS	
*P. aeruginosa*	Disc diffusion method	0.001 g/10 mL	Incubated at 37 °C for 24 h	18.5 mm zone of inhibition	Probably cell membrane disruption and oxidative stress from ROS	
*E. coli*	Disc diffusion method	0.001 g/10 mL	Incubated at 37 °C for 24 h	25.1 mm zone of inhibition	Probably cell membrane disruption and oxidative stress from ROS	
*S. aureus*	Disc diffusion method	0.001 g/10 mL	Incubated at 37 °C for 24 h	16.3 mm zone of inhibition	Probably cell membrane disruption and oxidative stress from ROS	[76]
*B. subtilis*	Disc diffusion method	0.001 g/10 mL	Incubated at 37 °C for 24 h	22.2 mm zone of inhibition	Probably cell membrane disruption and oxidative stress from ROS	
*P. aeruginosa*	Disc diffusion method	0.001 g/10 mL	Incubated at 37 °C for 24 h	34.5 mm zone of inhibition	Probably cell membrane disruption and oxidative stress from ROS	
*E. coli*	Disc diffusion method	0.001 g/10 mL	Incubated at 37 °C for 24 h	16.4 mm zone of inhibition	Probably cell membrane disruption and oxidative stress from ROS	
*B. subtilis* ATCC 6633	Agar plate well diffusion method	5 mg mL^−1^	Not indicated	15.6 mm zone of inhibition	Attributed to size effects	[3]
*S. aureus* ATCC 35556	Agar plate well diffusion method	5 mg mL^−1^	Not indicated	20 mm zone of inhibition	Attributed to size effects	
*P. aeruginosa* ATCC 10145	Agar plate well diffusion method	5 mg mL^−1^	Not indicated	11.3 mm zone of inhibition	Attributed to size effects	
*E. coli* ATCC 23282	Agar plate well diffusion method	5 mg mL^−1^	Not indicated	12 mm zone of inhibition	Attributed to size effects	
*B. subtilis* ATCC 6633	MIC and MLC	0.035–5 mg mL^−1^	Optical density (OD600) taken after incubation at 24 h	2.5 mg mL^−1^	Attributed to size effects	[3]
*S. aureus* ATCC 35556	MIC and MLC	0.035–5 mg mL^−1^	Optical density (OD600) taken after incubation at 24 h	5 mg mL^−1^	Attributed to size effects	
*P. aeruginosa* ATCC 10145	MIC and MLC	0.035–5 mg mL^−1^	Optical density (OD600) taken after incubation at 24 h	2.5 mg mL^−1^	Attributed to size effects	
*E. coli* ATCC 23282	MIC and MLC	0.035–5 mg mL^−1^	Optical density (OD600) taken after incubation at 24 h	2.5 mg mL^−1^	Attributed to size effects	
*E. coli*	Agar plate well diffusion method	2, 4, and 8 mg mL^−1^	Incubated at 37 °C for 24 h	23.5 mm zone of inhibition at a dose of 8 mg mL^−1^	Attributed to size effects and ROS damage to bacteria DNA, protein, and cell membrane	[7]
*Klebsiella pneumoniae*	Agar plate well diffusion method	2, 4, and 8 mg mL^−1^	Incubated at 37 °C for 24 h	27.2 mm zone of inhibition at a dose of 8 mg mL^−1^	Attributed to size effects and ROS damage to bacteria DNA, protein, and cell membrane	
*B. subtilis*	Agar plate well diffusion method	2, 4, and 8 mg mL^−1^	Incubated at 37 °C for 24 h	25.3 mm zone of inhibition at a dose of 8 mg mL^−1^	Attributed to size effects and ROS damage to bacteria DNA, protein, and cell membrane	
*Bacillus licheniformis*	Agar plate well diffusion method	8 mg mL^−1^	Incubated at 37 °C for 24 h	24.2 mm zone of inhibition at a dose of 8 mg mL^−1^	Attributed to size effects and ROS damage to bacteria DNA, protein, and cell membrane	
*E. coli*	Disc diffusion method	31.25–500 µg/mL	Incubated at 37 °C for 24 h	22.8 mm zone of inhibition at a dose of 500 µg/mL	Attributed to size effects and ROS effects on cellular contents	[78]
*P. aeruginosa*	Disc diffusion method	31.25–500 µg/mL	Incubated at 37 °C for 24 h	28.4 mm zone of inhibition at a dose of 500 µg/mL	Attributed to size effects and ROS effects on cellular contents	
*S. aureus*	Disc diffusion method	31.25–500 µg/mL	Incubated at 37 °C for 24 h	29.2 mm zone of inhibition at a dose of 500 µg/mL	Attributed to size effects and ROS effects on cellular contents	

**Table 4:** Cobalt oxide nanocomposites or other cobalt oxide based nanoparticles and bacteria remediation capability.

Material Used in Study	Target Bacteria in Study	Method of Assessing Activity on Bacteria	Concentration Used	Contact Time and Other Conditions	Antibacterial/Inhibitory Activity	Summary of Mechanism of Antibacterial/Inhibitory Activity	Reference
α-Fe_2_O_3_/Co_3_O_4_	*B. subtilis*	Disc diffusion method	400, 600 and 800 µg	Incubated at 37 °C for 24 h	21 mm zone of inhibition at a dose of 800 µg	Attributed to ROS effects on cellular contents	[50]
	*S. aureus*				24 mm zone of inhibition at a dose of 800 µg		
	*E. coli*				26 mm zone of inhibition at a dose of 800 µg		
	*S. typhi*				19 mm zone of inhibition at a dose of 800 µg		
α-Fe_2_O_3_/Co_3_O_4_	*B. subtilis*	Growth curve analysis	45, 60, 75, 90 and 120 mg/dL	Incubated at 37 °C for 24 h (reading taken at 6 h intervals)	OD600 = ∼0.3 at a concentration of 120 mg/dL after 24 h; MIC = 90 mg/dL	Attributed to ROS effects on cellular contents	[50]
	*S. aureus*				OD600 = 0 at a concentration of 120 mg/dL after 24 h; MIC = 75 mg/dL		
	*E. coli*				OD600 = 0 at a concentration of 120 mg/dL after 24 h; MIC = 45 mg/dL		
	*S. typhi*				OD600 = ∼0.01 at a concentration of 120 mg/dL after 24 h; MIC = 60 mg/dL		
β-CoMoO_4-_Co_3_O_4_	*E. coli*	Agar plate well diffusion method	1.56–50 mg/mL	Incubated at 37 °C for 24 h	17 mm zone of inhibition at a dose of 50 mg/mL	Electrostatic interactions with bacteria and ROS effects	[89]
	*P. aeruginosa*				19 mm zone of inhibition at a dose of 50 mg/mL		
	*S. aureus*				18 mm zone of inhibition at a dose of 50 mg/mL		
Co/Co_3_O_4_	*B. subtilis*	MIC and MBC	∼0–2000 µg/mL	CLSI guidelines	MIC = ∼125 µg/mL MBC = 2000 µg/mL	Not indicated	[51]
	*S. aureus*				MIC = ∼500 µg/mL MBC = 2000 µg/mL		
	*P. aeruginosa*				MIC = 31.25 µg/mL MBC = ∼500 µg/mL		
	*K. pneumonia*				MIC = ∼500 µg/mL MBC = 1000 µg/mL		
	*E. coli*				MIC = ∼500 µg/mL MBC = 1000 µg/mL		
Ni doped-Co_3_O_4_ (20 wt% of Ni)	*E. coli* MTCC 443	Agar plate well diffusion method	100 µg/mL	Incubated at 37 °C for 24 h	20 mm zone of inhibition	Attributed to interactions of nanoparticle with bacteria cell membrane	[39]
	*P. aeruginosa* MTCC 2453				14 mm zone of inhibition		
	*B. subtilis* MTCC 441				18 mm zone of inhibition		
	*S. aureus* MTCC 96				13 mm zone of inhibition		
Co_3_O_4_@ZrO_2_	*E. coli*	Agar plate well diffusion method	50, 100 and 200 µg/mL	Incubated at 37 °C for 24 h	∼˂1 mm zone of inhibition at a dose of 200 µg/mL	Attributed to cell wall penetration and genotoxicity resulting in cell deformation	[53]
	*P. aeruginosa*				∼13 mm zone of inhibition at a dose of 200 µg/mL		
	*B. subtilis*				∼1 mm zone of inhibition at a dose of 200 µg/mL		
	*S. aureus*				∼12 mm zone of inhibition at a dose of 200 µg/m		

## Data Availability

Not applicable.

## Abbreviations

AFM	Atomic Force Microscopy
CLSI	Clinical and Laboratory Standards Institute
CV	Cyclic voltammetry
DLS	Dynamic light scattering
DNA	Deoxyribonucleic acid
DRS	Diffuse reflectance spectroscopy
DTA	Differential thermal analysis
EDS	Energy-dispersive x-ray spectroscopy
FESEM	Field emission scanning electron microscope
Fluor	Fluorescent spectroscopy
FTIR	Fourier transform infrared spectroscopy
HRSEM	High-resolution scanning electron microscopy
HRTEM	High-resolution transmission electron microscopy
IR	Infra-red spectroscopy
MBC	Minimum bactericidal concentration
MIC	Minimum inhibitory concentration
MLC	Minimum lethal concentration
PL	Photoluminescence spectroscopy
PSA	Particle size analysis
Raman	Raman spectroscopy
ROS	Reactive oxygen species
SEM	Scanning electron microscopy
TEM	Transmission electron microscopy
TGA	Thermogravimetric analysis
UNEP	United Nations Environment Program
UV	Ultraviolet spectroscopy
UV-Vis	Ultraviolet-visible spectroscopy
UV-Vis-NIR	Ultraviolet-visible-near-infrared spectroscopy
VSM	Vibrating sample magnetometry
WHO	World Health Organization
XPS	X-ray photoelectron spectroscopy
XRD	X-ray diffraction spectroscopy
